# A Rare Case of Transepiphyseal Distal Femur Fracture Dislocation With Delayed Recovery of a Common Peroneal Nerve Injury

**DOI:** 10.7759/cureus.65318

**Published:** 2024-07-24

**Authors:** Dattatray Bhakare, Amit Patil, Rahul Salunkhe, Swati Bhakare, Rachit Mitra

**Affiliations:** 1 Orthopaedics and Trauma, Dr. D. Y. Patil Medical College, Hospital and Research Centre, Dr. D. Y. Patil Vidyapeeth, Pune (Deemed to Be University), Pune, IND; 2 Obstetrics and Gynaecology, Dr. D. Y. Patil Medical College, Hospital and Research Centre, Dr. D. Y. Patil Vidyapeeth, Pune (Deemed to Be University), Pune, IND

**Keywords:** peroneal nerve palsy, foot drop, epiphyseal fracture, k-wiring, neurological complication, neurovascular compression, pediatric fractures, acute trauma care

## Abstract

The aim of this study is to bring attention to a unique case and our approach to treatment in this context. We describe a case of an 11-year-old male who presented to us with an injury to his left knee following trauma with pain, swelling, shortening and deformity for one day. An X-ray revealed a transepiphyseal fracture dislocation of the left distal femur (Salter-Harris type 1 injury) and neurovascular examination was conclusive of foot drop which pointed towards injury to common peroneal nerve (CPN).

The patient was taken up for closed reduction with percutaneous pinning under mobile C-arm guidance. The fracture was reduced and fixed with two cross K-wires and immobilized with the above knee anterior-posterior slab for six weeks. The wires were removed after six weeks but there was no improvement in the dorsiflexion of the left ankle. An electromyography (EMG) and nerve conduction velocity (NCV) study test was performed after 12 weeks which showed decreased amplitude and prolonged latency in the left CPN with early denervation of the muscles supplied by the left CPN. Fifteen weeks of follow-up showed complete recovery in the dorsiflexion of the left ankle with a slight lag in the extension of the left great toe making this an unusually delayed recovery of CPN palsy following a distal femur transepiphyseal fracture.

## Introduction

Distal femoral physeal fracture injuries comprise 2% of all physeal injuries [1]. Complications after initial treatment from these injuries range from 40% to 60%. The acute complications are neurovascular injuries and long-term complications include growth disturbances, leg length discrepancies, angular deformities and growth arrest [1]. Hence, the management of these fractures appropriately for long-term success is important.

Salter-Harris classification divides the injury in children into five parts and grades fractures of the growth plate according to the involvement of adjacent metaphysis and epiphysis which is based on radiographic appearance, mechanism of injury and prognosis, concerning the growth disturbances [2,3].

Common peroneal nerve (CPN) injury is the most common neuropathy of the lower extremity and the third most common neuropathy overall, after median and ulnar nerve neuropathy [4]. Due to its close proximity and superficial location, it is susceptible to traumatic and entrapment injuries [5]. History and clinical examination can be aided with a plain radiograph when CPN injury is suspected. The nerve injuries can be broadly divided into neuropraxia, axonotmesis and neurotmesis. Electrodiagnostic studies such as electromyography (EMG) and nerve conduction velocity (NCV) tests are helpful in diagnosing CPN palsy. These tests assess the motor and sensory axons of the CPN and its branches. They also help to localize the nerve injury. These tests are especially useful in patients with known cases of foot drop due to peroneal nerve injury to assess the prognosis and to plan long-term management post-operatively.

## Case presentation

This is a case of an 11-year-old child who presented to us in the emergency room with injury to the left knee with complaints of pain which was acute in onset, aggravated on movement and relieved by pain medication and immobilization. There was a history of trauma on the left knee where his left knee was in an extended position following which he was hit by a two-wheeler resulting in a hyperextension injury. On examination, diffuse tenderness and a palpable mass were present in the distal femur with a restricted range of motion. Upto 6 cm shortening was present and the attitude of the limb was in hyperextension. Distal pulses were palpable but dorsiflexion of the left ankle was absent. The patient directly came to our hospital where he was treated conservatively with an above-knee slab and pain medication during the first few hours.

**Figure 1:**
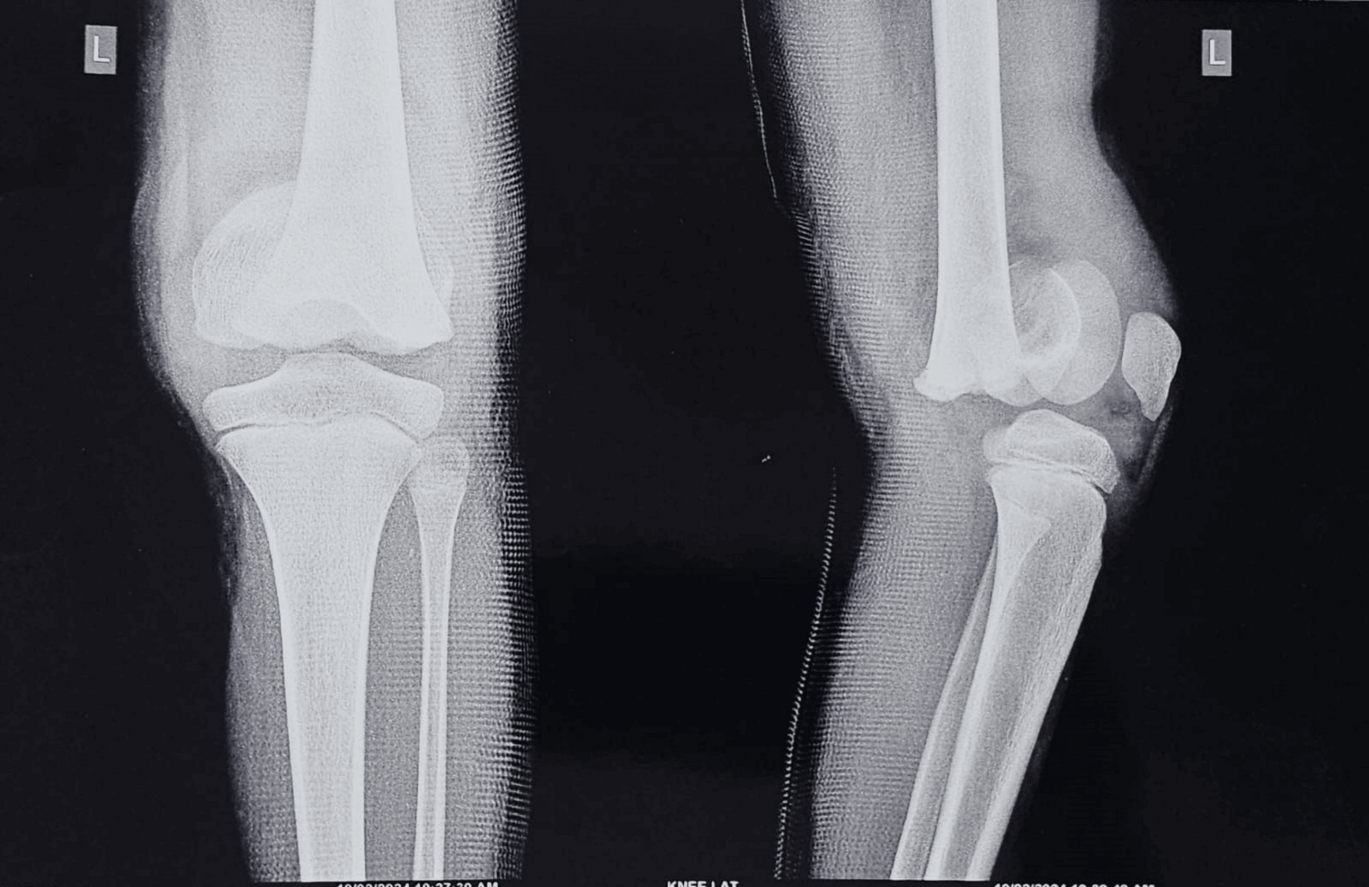
Pre-operative X-ray showing the left distal femur transphyseal fracture.

An X-ray revealed a distal femur completely displaced Salter-Harris type 1 transepiphyseal injury (Figure 1).

All routine laboratory and metabolic profiles performed were within normal ranges. The patient was taken up for closed reduction with percutaneous pinning under general anaesthesia six hours after admission following the pre-anaesthesia check-up.

**Figure 2:**
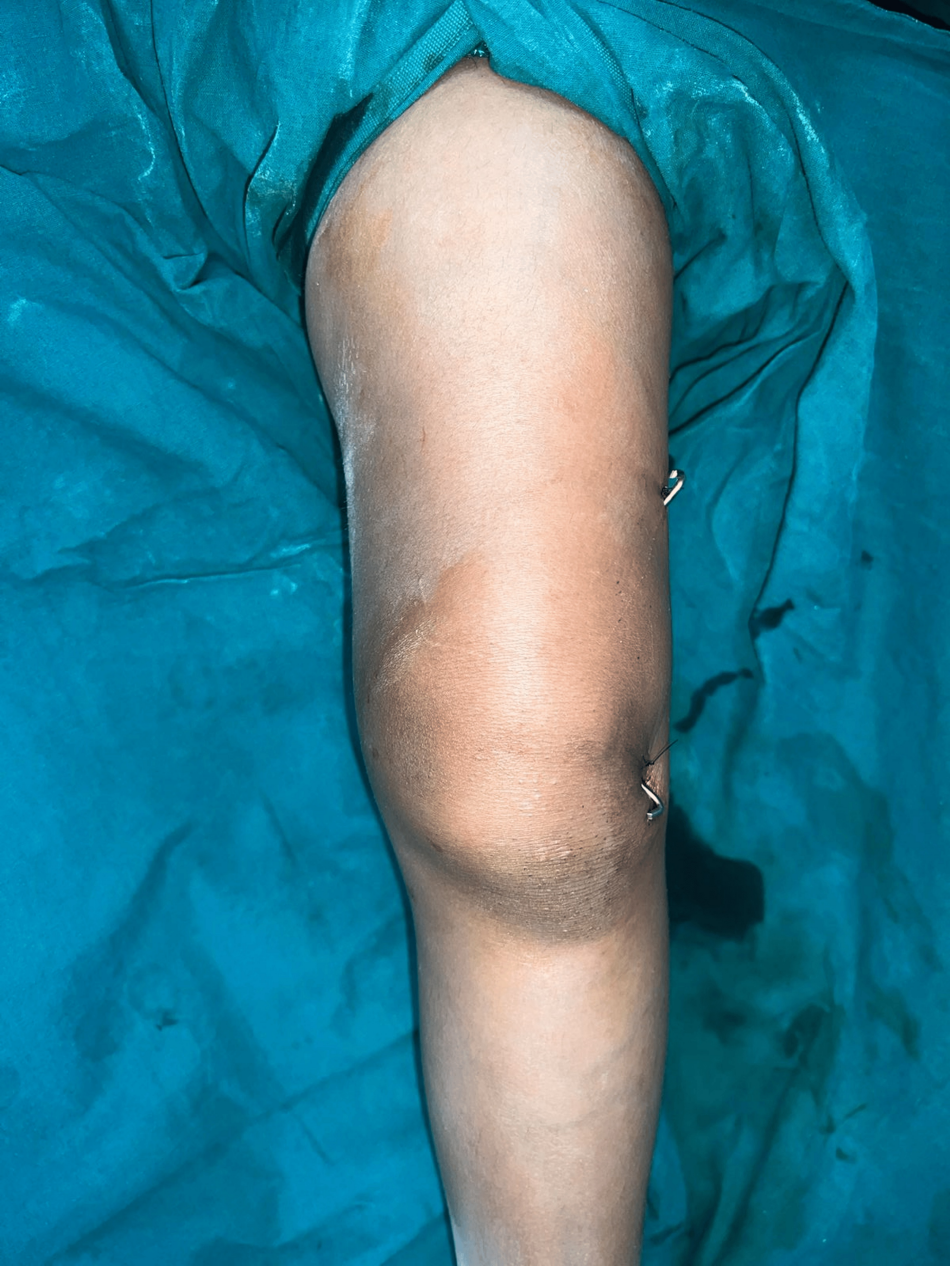
Clinical picture showing two 2 mm crossed K-wires passing from lateral to medial side.

Closed reduction with gentle traction was achieved, followed by gradual flexion of the knee (Wilkin’s manoeuvre). The physeal fragment was reduced with both thumbs, and reduction was checked under mobile C-arm guidance. Final fixation was achieved with percutaneous two smooth Kirschner (K) wires of 2 mm in a cross construct as shown in Figure 2.

**Figure 3:**
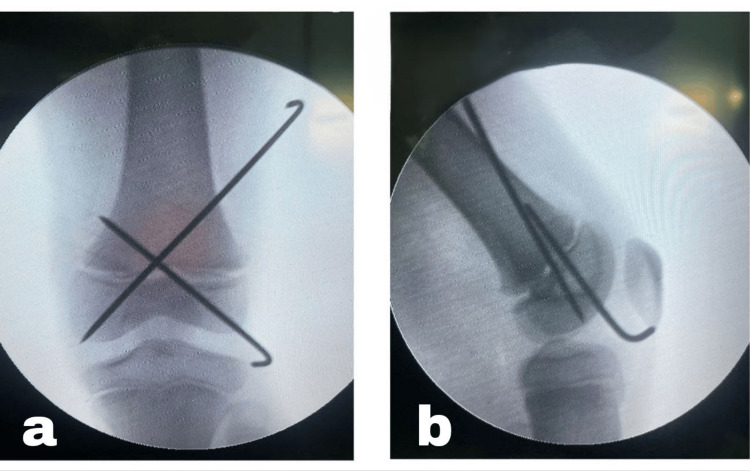
Distal femoral transphyseal fracture after closed reduction and percutaneous pinning: (a) anteroposterior view; (b) lateral view.

The reduction was confirmed under C-arm guidance as shown in Figure 3.

**Figure 4:**
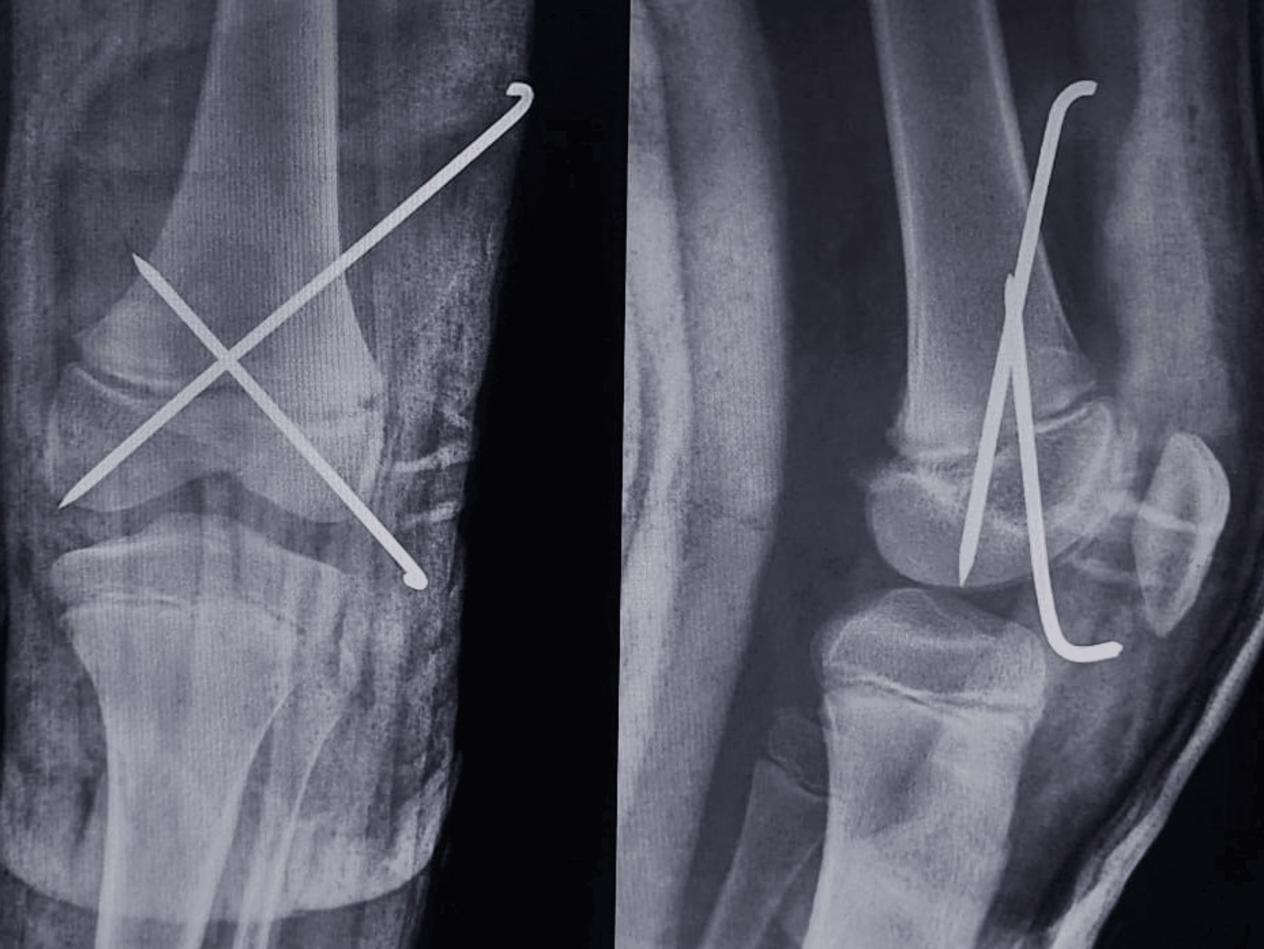
Post-operative X-ray showing anteroposterior and lateral views of the knee joint.

After pinning, an above-knee slab posteriorly and anteriorly across the knee joint was given and ankle dorsiflexion was absent postoperatively. Post-op X-ray (Figure 4) shows a reduction of fracture dislocation with two cross K-wires passed from the lateral to the medial side.

The patient was immobilized for six weeks with an above-knee slab anteriorly and posteriorly and kept non-weight bearing. The patient was called for follow-up after six weeks where his K-wires were removed and a gradual knee range of motion was performed. As the patient had a foot drop on the affected side, weight-bearing was not advised. There was no ankle dorsiflexion present after six weeks. An EMG NCV test was done after 12 weeks postoperatively which showed absent response in the left peroneal nerve and decreased amplitude and prolonged latency in the left CPN. Needle EMG was suggestive of changes of early denervation of the muscles supplied by the left CPN with no evidence of reinnervation. Neurologic examination revealed the slight flickering movement of the dorsiflexor of the left ankle after 12 weeks postoperatively.

**Figure 5:**
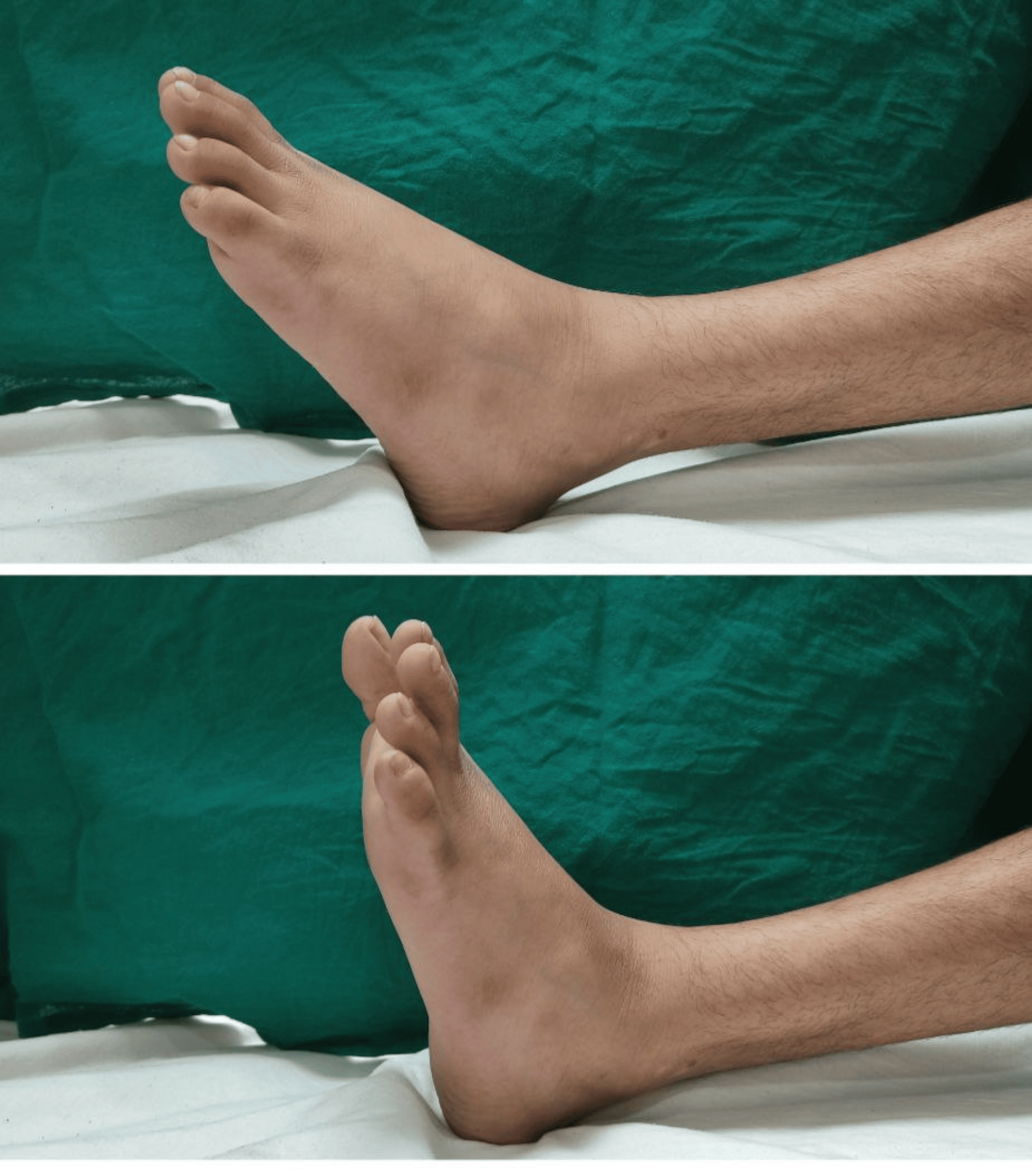
The figure showing dorsiflexion of the left ankle after 15 weeks.

The patient was followed up after 15 weeks. He had a complete return of ankle dorsiflexion with a slight lag in the extension of the great toe and a full knee range of motion was achieved (Figure 5).

**Figure 6:**
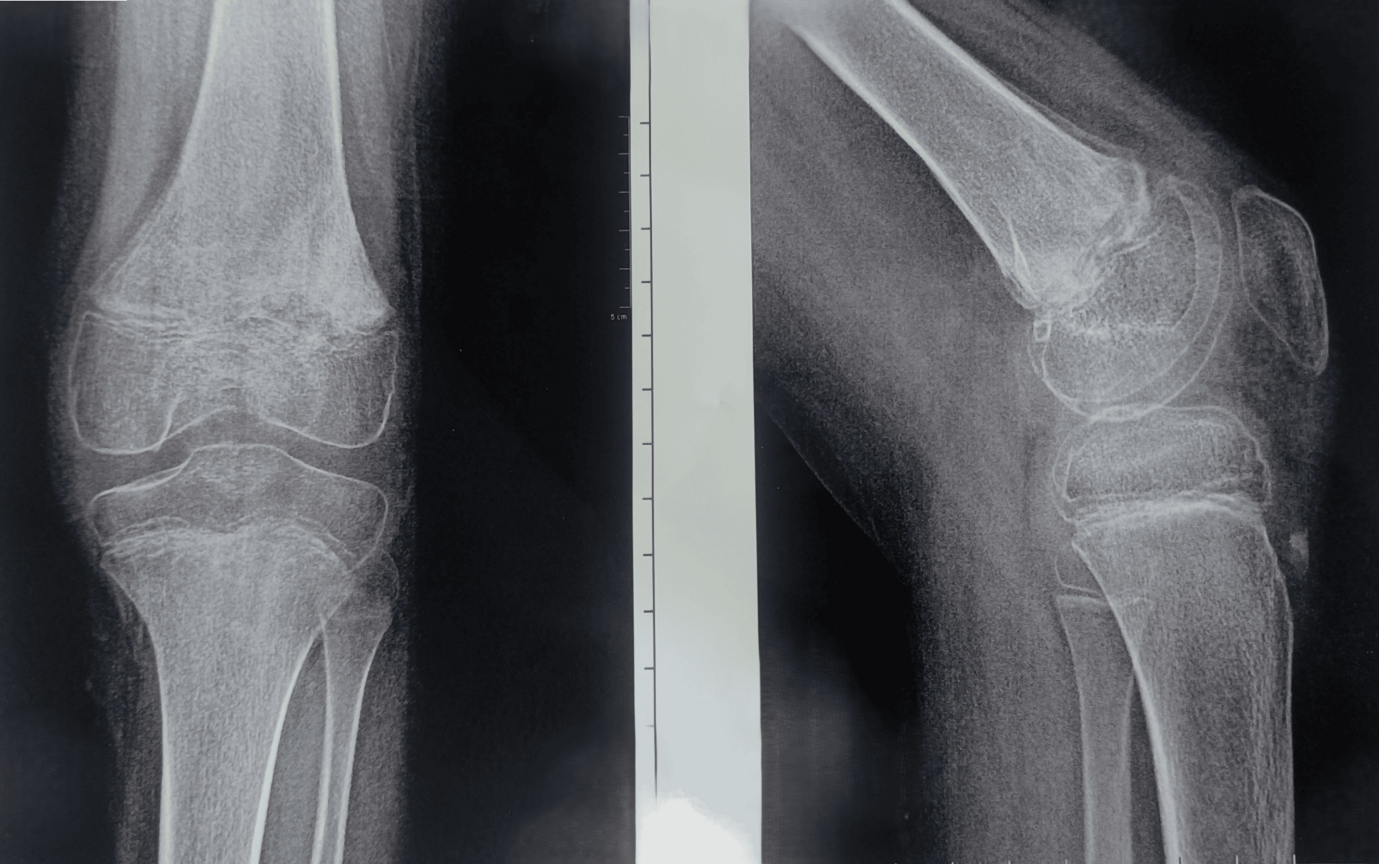
Post-operative X-ray after 15 weeks

The patient was weight-bearing and the post-op X-ray after 15 weeks showed no abnormality (Figure 6).

## Discussion

Fracture of the distal femur comprises 2% of all physeal injuries making this an uncommonly injured site [1]. Knowing how to manage these fractures in an appropriate timely manner can prevent complications (which are quite common for 40-60%) and lead to better long-term positive outcomes [1]. Earlier, distal femoral epiphyseal slip patients presenting acutely were managed with only closed reduction and casting under general anaesthesia. However, in a high-velocity type of injury, only closed reduction and casting are not adequate, as the chances of displacement with such treatment are reported to be 43-60% [6]. Closed reduction with percutaneous pinning aided with casting is required to counteract the strong muscular forces acting across the joint [7] thus reducing the chances of future displacement, which was exactly what was done in our case. Peroneal nerve injury has been a rare complication following a Salter-Harris type 1 distal femur transepiphyseal fracture dislocation, making our case unique. The risk of neurological injury associated with a distal femoral transepiphyseal slip is recognized in the literature with CPN injury incidence being 2-7% [8]. A review of 206 patients with distal femoral physeal fracture conducted by Beaty and Kumar [9] showed a 2% incidence (four patients) of neurovascular injury, injuries being mostly vascular with no patients cited for associated peroneal nerve injury. In a review of 311 patients with distal femoral physeal fracture, it was found that a total of six cases (2%) showed peroneal nerve palsy [8]. Hence, the literature on CPN palsy following a distal femur fracture is limited and most of them showed early recovery.

In our case, an 11-year-old male presented to us with an injury to the left knee following a traumatic event, the mechanism of injury being hyperextension type, following which he developed pain and swelling. Detailed clinical examination revealed a 6 cm shortening of the affected left limb and absent dorsiflexion of the left ankle most probably due to traction injury to the CPN. This necessitates a detailed examination of all physeal fracture patients who present to us in an acute manner so that such important details should not be missed out to prevent a long-term deformity and to manage these cases promptly.

An X-ray was done which showed a left distal trans-physeal femur fracture dislocation of Salter-Harris type 1. The patient was taken for closed reduction with percutaneous pinning after an unremarkable pre-anaesthesia check-up. In such cases, care has to be taken that the fracture has been reduced properly which was done in our case with traction and manipulation (Wilkin’s manoeuvre) under C-arm guidance. Two 2 mm K-wires were drilled in a cross manner to hold the reduction. It's important to pass the K-wire in such a manner that it does not hamper the joint line and crosses from lateral to medial away from the CPN and without multiple attempts which was exactly what was done in our case. The reduction was found to be satisfactory and it was protected with an above knee anterior posterior slab for six weeks. The cast was not given in our case because of marked swelling.

The patient was advised non-weight-bearing walking with a walker. The patient was called for follow-up after six weeks and both the K-wires and slab were removed which is consistent with other literature where K-wires were removed from a period of four to six weeks followed by a complete range of motion of knee and ankle. On examination, there was an absent dorsiflexion of the left ankle. This is in contrast to the studies conducted by Sloboda et al. [8] which showed a complete return of dorsiflexion of the ankle and extension of the great toe by six weeks. Hence, our case becomes a rare case where there was a delayed recovery of the peroneal nerve palsy.

The patient was called for follow-up at 12 weeks and there was flickering movement of the left ankle dorsiflexor. An EMG NCV test was performed which showed an absent response in the left CPN and decreased sensory nerve action potential (SNAP) amplitude and prolonged latency in the left CPN. Peroneal nerve injury associated with distal femur fracture appears to be mostly neuropraxia or axonotmesis type of injury which recovers spontaneously without intervention. In our case, after 12 weeks, there was flickering of the ankle joint hence making this a delayed recovery of CPN palsy.

At 15 weeks of follow-up, the patient had achieved full dorsiflexion of the left ankle with a slight lag in the extension of the great toe with the full range of motion on the knee joint. This proves that the palsy of the peroneal nerve associated with distal femur transepiphyseal fracture was neuropraxia which showed a delayed recovery. Such types of injuries should be managed with patience and usually, exploration of the nerve is not necessary.

## Conclusions

Peroneal nerve palsy is a rare complication associated with distal femur physeal fractures. Due to their superficial nature, it is prone to injury due to any traumatic event. Most injuries are neuropraxia or axonotmesis type of injury which recover spontaneously without any intervention within four to six weeks but in our case there was an unusual delayed recovery of almost 15 weeks. Detailed careful neurovascular examination at the time of admission is of utmost importance. The most appropriate management of distal femur Salter-Harris type 1 transepiphyseal fracture dislocation is closed reduction and cross K wiring. Our study proves that exploration of the nerve can be delayed and CPN palsy should be managed conservatively and with patience.
